# Detecting primitive hematopoietic stem cells in total nucleated and mononuclear cell fractions from umbilical cord blood segments and units

**DOI:** 10.1186/s12967-015-0434-z

**Published:** 2015-03-18

**Authors:** John Patterson, Cally H Moore, Emily Palser, Jason C Hearn, Daniela Dumitru, Holli A Harper, Ivan N Rich

**Affiliations:** Beth-Ell College of Nursing and Health Science, University of Colorado at Colorado Springs, Colorado Springs, Colorado USA; Department of Biological Engineering, University of Colorado, Boulder, Colorado USA; Department of Biology, University of Colorado at Colorado Springs, Colorado Springs, Colorado USA; HemoGenix, Inc, 1485 Garden of the Gods Road, Suite 152, Colorado Springs, CO 80906 USA

**Keywords:** Colony-forming unit, ATP bioluminescence, Proliferation assay, Umbilical cord blood, Stem cell transplantation, Total nucleated cell fraction, Stem cell processing, Viability, Segment, Umbilical cord blood unit

## Abstract

**Background:**

Rare hematopoietic stem cell populations are responsible for the transplantation engraftment process. Umbilical cord blood (UCB) is usually processed to the total nucleated cell (TNC), but not to the mononuclear cell (MNC) fraction. TNC counts are used to determine UCB unit storage, release for transplantation and correlation with time to engraftment. However, the TNC fraction contains varying concentrations of red blood cells, granulocytes, platelets and other cells that dilute and mask the stem cells from being detected. This does not allow the quality and potency of the stem cells to be reliably measured.

**Methods:**

63 UCB segments and 10 UCB units plus segments were analyzed for the response of both primitive lympho-hematopoietic and primitive hematopoietic stem cells in both the TNC and MNC fractions. The samples were analyzed using a highly sensitive, standardized and validated adenosine triphosphate (ATP) bioluminescence stem cell proliferation assay verified against the colony-forming unit (CFU) assay. Dye exclusion and metabolic viability were also determined.

**Results:**

Regardless of whether the cells were derived from a segment or unit, the TNC fraction always produced a significantly lower and more variable stem cell response than that derived from the MNC fraction. Routine dye exclusion cell viability did not correspond with metabolic viability and stem cell response. Paired UCB segments produced highly variable results, and the UCB segment did not produce similar results to the unit.

**Discussion:**

The TNC fraction underestimates the ability and capacity of the stem cells in both the UCB segment and unit and therefore provides an erroneous interpretation of the of the results. Dye exclusion viability can result in false positive values, when in fact the stem cells may be dead or incapable of proliferation. The difference in response between the segment and unit calls into question the ability to use the segment as a representative sample of the UCB unit. It is apparent that present UCB processing and testing methods are inadequate to properly determine the quality and potency of the unit for release and use in a patient.

## Introduction

Hematopoietic stem cell transplantation using bone marrow, mobilized peripheral blood or umbilical cord blood (UCB) as stem cell sources, are routine clinical procedures. Yet the presence and functionality of the stem cells is mostly assumed, rather than actually measured. The methylcellulose colony-forming unit (CFU) assay has been used to detect many different cell populations from stem cells with high proliferative potential [1-4] to precursor cells that demonstrate few cell divisions [5,6]. Although the assay is not routinely used in bone marrow or mobilized peripheral blood stem cell transplantation processing [7], a functional assay is routinely required for cord blood processing, since UCB units are cryopreserved and engraftment occurs later than that for bone marrow or mobilized peripheral blood [8,9]. However, rather than detecting stem cells, the CFU assay is usually employed to detect granulocyte-macrophage (GM) progenitor cells as an indicator of time to neutrophil engraftment [10]. With the exception of CD34 enumeration, which became routine in the early 1990s [11], the CFU assay together with total nucleated cell (TNC) counts and viability represent the three basic tests that have been continuously used to characterize UCB cells for storage and transplantation purposes since the first UCB transplant in 1988 [12].

Since its introduction in 1966 for murine cells [13,14], and later for human bone marrow cells [15], counting colonies in a methylcellulose CFU assay has been the method of choice to determine primitive hematopoietic cell functionality. However, both clonal and liquid culture assays have been reported using an instrument-based MTT (3-(4,5-dimethylthiazol-2-yl)-2,5,-diphenyltetrazolium bromide) colorimetric readout, based on the reduction of the tetrozolium substrate by the mitochondria to a yellow formazan product. This provides a metabolic viability version of the CFU assay [16-18]. The ability to use an instrument-based, biochemical readout, such as MTT, laid the groundwork for combining the methylcellulose clonal CFU assay with an adenosine triphosphate (ATP) marker for measuring in vitro hematopoietic stem and progenitor cell proliferation ability. This was demonstrated in 2005 [19], and later used to evaluate umbilical cord blood progenitor cells [20].

Adenosine triphosphate is the cell’s source of chemical energy. It is produced in the mitochondria of cells. Hepatocytes and kidney cells for example, have inherently high levels of ATP associated with their high levels of metabolism. Other cells, such as quiescent stem cells, exhibit low levels of metabolism and therefore have low basal levels of ATP production. Cells require ATP for numerous biochemical reactions, from cellular respiration to DNA synthesis and cell division. During these reactions, ATP is reduced to adenosine di- (ADP) and monophosphate (AMP) and the high-energy phosphate atoms are recycled to produce more ATP. It follows that ATP is vital to the cell’s metabolism and health; cells that do not or cannot produce ATP are metabolically dead.

Crouch et al. introduced the use of ATP bioluminescence measurement for proliferating cells in 1993 [21]. The reaction requires the addition of luciferin and the enzyme luciferase to produce oxyluciferin, which creates a bioluminescence signal that is measured as photons by a luminometer. To measure intracellular ATP (iATP) in cells, the ATP must be the limiting factor. The reaction also requires the presence of oxygen. Red blood cells (RBC), although they do not contain mitochondria, have both high levels of ATP and oxygen. If ATP is being used to measure cell proliferation in suspensions containing high concentrations of RBCs, the latter acts as an impurity in the cell preparation producing false positive results. Simply removing or reducing the RBCs can negate the problem. However, if the RBCs are lysed, the presence of hemoglobin can inhibit the ATP luciferin/luciferase reaction and cause low ATP concentrations. The presence of RBCs could therefore pose a problem to characterize rare stem cell populations in hematopoietic cell therapy products that use a total nucleated cell (TNC) fraction. This is because RBCs and other cell impurities, including granulocytes, platelets and other cell types, can be present in varying amounts depending on the processing method [22].

Although it was shown that UCB units could be processed by density gradient centrifugation to produce large numbers of mononuclear cells (MNCs) [23,24], the TNC fraction is the product of choice. It is more rapidly produced than the MNC fraction and is less costly. Over the years, the TNC and CFU counts, number of CD34^+^ cells and even viability have all been associated, to varying degrees, with clinical outcome [10,25,26]. In addition, these and especially TNC, are used as decision-making parameters to permanently store UCB units for transplantation purposes and select and release a unit for use in a patient.

One of the major hurdles in stem cell transplantation has been the standardization, optimization and validation of procedures and assays [27,28] required to produce a stem cell product that can be accurately and reliably characterized prior to use. Numerous articles have addressed issues such as processing procedures, cryopreservation techniques, pre-freeze and post-thaw methodologies. Nevertheless, viability, CD34, the CFU assay and clinical outcome are based on the TNC fraction. Therefore, despite the problems associated with a TNC fraction to detect and measure the presence of stem cells, we tested a “null hypothesis” by assuming that UCB segments and units did not have to be further purified in order to reliably and reproducibly measure stem cells in a standardized and validated assay. In short, measurement of UCB stem cells should not be dependent upon the purity of the UCB preparation. The results presented below reject that hypothesis and call into question UCB quality and potency based on the TNC fraction.

## Materials and methods

### Umbilical cord blood samples

Umbilical cord blood sample segments used in this study were purchased from two different cord blood banks as cryopreserved research samples. Cord blood bank (CBB) 1 processed samples using Sepax technology (BioSafe America), while CBB 2 employed AXP technology (Cesca Therapeutics, formerly ThermoGenesis, Rancho Cordova, CA). In addition, individual units with attached segments were also obtained. No pre-freeze samples were available for these studies. Donors gave their consent for the cells to be used for research purposes. The segment volume ranged from 0.2 mL to less than 0.1 mL. The unit volumes were between 20 mL to 30 mL each.

### Preparation of samples for use

Umbilical cord blood segments and units were both thawed in a 37°C water bath. After thawing a segment, a 1 mL syringe attached to a 22 gauge needle was used to puncture two holes in the top of the segment and the contents gently mixed before removing the cells. The volume was noted and the contents transferred to a 2 mL tube containing the same volume of Iscove’s Modified Dulbecco’s Medium (IMDM, Life Technologies). The contents were gently mixed, but a wash step was not performed. This would be a normal procedure if cells from a segment were tested, since the number of cells available would be reduced by a wash step. However, for some of the segments, a small volume was removed, without any additional treatment, to perform a differential cell count (Medonic CA620, Stockholm, Sweden), viability using 7-aminoactinomycin D (7-AAD) by flow cytometry (Accuri C6, BD Biosciences) and nucleated cell count (Z2 particle counter, Beckman Coulter). This post-thaw, unseparated fraction was designated the TNC fraction. The remaining cells were under-layered with 1.0 mL of NycoPrep 1.077 (Axis Shield, Oslo, Norway) density gradient medium and centrifuged according to the manufacturer’s instructions. The interface cells were removed and transferred to another tube. This separated cell fraction was designated the MNC fraction, which was resuspended in 2.0 mL of IMDM, centrifuged at 200 × g for 10 minutes at room temperature to wash the cells. The resulting cell pellet was resuspended in 0.2 mL of IMDM. A second nucleated cell count, viability, and for some samples, a differential cell count was performed. Umbilical cord blood units were handled and processed in exactly the same manner, except that the contents of the units were allowed to drain into a 50 mL tubed through one of the ports, mixed gently and aliquoted into 0.3 mL samples and transferred to 2 mL tubes for processing after several samples had been removed to obtain initial differential and nucleated cell counts and viability values. Each 0.3 mL aliquot was diluted with 0.3 mL of IMDM and fractionated using the same procedure as the segments.

### Colony-Forming Unit (CFU) assay

A miniaturized CFU assay (CAMEO-4, HemoGenix, Colorado Springs, CO) was performed as previously described [29]. The cell concentration was adjusted to 0.5 × 10^6^ cells/mL and a 3-point cell dose response prepared to produce final concentrations. The total volume prepared was 0.6 mL, including cells. Using a positive displacement electronic pipette (Eppendorf, Hamburg, Germany), 0.1 mL was dispensed into 4 replicate wells in a 35 mm Petri dish, each with a growth surface area of 0.95 mm^2^. The cell population detected was the primitive hematopoietic stem cell or colony-forming unit – granulocyte, erythroid, macrophage, megakaryocyte (CFC-GEMM) stimulated with erythropoietin (EPO), granulocyte-macrophage colony stimulating factor (GM-CSF), thrombopoietin (TPO), stem cell factor (SCF), Flt-3 ligand and interleukins 3 and 6 (IL-3, IL-6). Cells were cultured in a 37°C humidified incubator gassed with 5% CO_2_ and 5% O_2_ [29]. Colonies were manually counted using an inverted microscope (40 – 100× magnification, Zeiss, U.S.A.) after 9–10 days of incubation to ensure that the colonies do not grow together. This is a shorter incubation period than normal for CFU cultures (14–16 days) and is primarily due to a difference in methylcellulose culture reagent formulation and the smaller growth surface area than for a 35 mm Petri dish.

### ATP Bioluminescence proliferation assay (assay performed in 96 plate)

Since the CFU assay cannot be used to directly measure the proliferation of stem cells, a 96-well plate, ATP bioluminescence assay was employed (HALO, HemoGenix, Colorado Springs, CO). This has been described in detail elsewhere [19,20,30]. In essence, when cells are stimulated to proliferate, their intracellular ATP concentrations increases several fold above the basal iATP level. The change in iATP concentrations directly correlates with the metabolic activity (viability) of the cells and their proliferation status. After cell culture, the cells are lysed to release iATP, which reacts with a luciferin/luciferase reagent to produce bioluminescence. This is detected as light in a luminescence plate reader. The assay does not incorporate methylcellulose and therefore is not a clonal assay. Instead, cells are grown and expanded in suspension that not only allows increased accuracy and sensitivity, but more rapid assay completion. Although the assay can be performed in just 5 days with high cell concentrations (2,500, 5,000 and 7,500 cells/well), a more optimal 7 day incubation time period using lower cell concentrations (1,000, 2,000 and 4,000 cells/well) was used in the majority of experiments described. As shown, this extra 2-day incubation produces an approximate 2–3 fold increase in ATP concentrations and therefore a concomitant increase in assay sensitivity.

Prior to measuring any samples, the ATP bioluminescence stem cell proliferation assay was calibrated using ATP calibrators and standardized using an external ATP standard curve. This allowed the non-standardized readout in relative luminescence units (RLU) to be interpolated as standardized ATP concentrations (μM). The ATP standard curve is a series of five ATP dilutions (from 0.01 μM to 1 μM) that produces a straight-line curve that can be fitted by linear regression analysis. The slope of the line must lie within specific upper and lower limits. The ATP calibrators are known ATP concentrations that must lie on the ATP standard curve and also produce values within specific upper and lower limits. When these conditions have been met, not only has an internal proficiency test been performed to ensure that the assay is working correctly, but also allows results from different experiments to be directly compared without normalization. In addition, the inclusion of these steps allowed the assay to be validated [31,32] according to regulatory guidelines [33].

All studies of UCB cell proliferation measured two stem cell populations. Since the ATP proliferation assays were methylcellulose-free and therefore not clonal, the first stem cell population was the equivalent to, and designated as, CFC-GEMM, using exactly the same growth factor cocktail as described for the CFU assay. The second was the more primitive lympho-hematopoietic high proliferative potential – stem and progenitor cell (HPP-SP), which included, interleukins 2 and 7 (Il-2, IL-7). For these studies, all growth factors and cytokines were obtained from CellGenix (Freiburg, Germany) and EPO was obtained from Cell Sciences (Canton, MA, USA). For both of the stem cell populations, two parameters of cell proliferation were measured simultaneously. The first was proliferation ability or status. It determines the amount of cell proliferation at a specific cell dose and point in time. The second parameter was proliferation potential, which is measured by the slope of the cell dose response linear regression [34]. The more primitive a stem cell population the greater the slope of the cell dose response and therefore the greater the proliferation potential of the stem cell. Measuring stem cell proliferation potential is also the basis for measuring stem cell potency [31,32,35-37].

### Statistics

Based on historical UCB sample data [32,35], a power calculation was used to determine a minimum sample size needed for the present studies (Systat Software, Version 13.1, San Jose, CA). For a power of 0.9, a total of 11 samples were required to reject our “null hypothesis”. In fact, a total of 63 individual cord blood segments obtained from two different cord blood banks were tested. Studies were initiated on 22 segment samples cultured for 5 days at high cell concentrations. The remaining 41 segments were tested at lower cell concentrations for 7 days. The standardized ATP assay allowed all of the results to be compared directly. In addition, several of the segments were provided in duplicate allowing a comparison not only between individual segments from the same lot (unit), but also to determine if differences might be due to methodological changes performed by the investigators. To determine if methodology might be a contributing factor to differences observed between segments, a Bland-Altman statistic was used. For the CFU assay, each data point was performed in quadruplicate and results calculated as the mean ± standard deviation. For the ATP proliferation assay, 8 replicates were performed for each data point. All of the results from these assays are based on raw data and are expressed as the mean ± standard deviation (SD) or standard error of the mean (SEM), where appropriate. In addition, the percent coefficients of variation (%CV) were also evaluated. To determine the slope of the cell dose response, linear regression analysis was performed given by parameter B in the equation Y = A + Bx, where A is the intercept with the ×-axis. For linear regression analysis, the slope and the correlation coefficient (r) were calculated. The goodness of fit (R^2^) was omitted since this can be calculated from the square root of the correlation coefficient. For all results demonstrating cell proliferation, the historical acceptance/rejection limit was 0.04 μM ATP ± 15%. This is provided in all applicable graphs. Cells exhibiting ATP values greater than 0.04 μM are usually capable of sustaining proliferation. Cell exhibiting ATP values within or just below the acceptance/rejection level will have limited proliferation capability, while cells showing ATP values of 0.01 μM or less will be metabolically dead and will not proliferate. Depending on whether a comparison was made between two groups or multiple groups, a two-tailed t-test or two-way analysis of variance (ANOVA) was performed, respectively. This and all other statistical analyses, with the exception of power analysis, used GraphPad Prism software (version 6f for Mac).

## Results

### Cord blood component parameters before and after fractionation

Table 1, shows the mean values of the basic cord blood components from 56 untreated (TNC fraction) and treated (MNC fraction) segments. Although cryopreservation results in lysis and cell death of many cell components, a significant proportion of mature cells remain post-thaw. Preparation of a MNC fraction further reduces these components and it would appear that a significant proportion of the components that make up the MNC fraction are removed or are lost from the TNC fraction. It might be assumed that this reduction also occurs for the stem cells. With a recovery of 30% or less, density gradient centrifugation is one of the least efficient cell purification methods. Nevertheless, as shown in the results below, the cell impurities in the post-thaw, TNC fraction mask and impair the stem cells to such an extent that it is difficult to accurately and reliably assess their presence and functional potential.

**Table 1:** **Combined percent reduction of mature blood components between TNC and MNC fractions for 56 segment samples**

**Segment cell composition**	**TNC fraction (Mean ± St.Dev)**	**MNC fraction (Mean ± St.Dev)**	**Percent reduction (Mean ± St.Dev)**
RBCs (× 10^12^/L)	0.74 ± 0.19	0.19 ± 0.09	74.32 ± 16.75
Hematocrit (%)	12.21 ± 0.034	1.52 ± 0.02	87.55 ± 20.45
Platelets (× 10^9^/L)	258.84 ± 124.04	15.34 ± 14.79	94.07 ± 5.25
White blood cells (× 10^9^/L)	20.04 ± 7.48	2.44 ± 3.00	87.82 ± 12.26
Granulocytes (× 10^9^/L)	2.30 ± 1.22	0.11 ± 0.03	95.22 ± 1.28
Lymphocytes (× 10^9^/L)	14.64 ± 5.51	1.72 ± 1.16	88.25 ± 10.34
Viability (%)	95.80 ± 1.13	99.76 ± 0.07	2.54

### The CFU differentiation assay and correlation with the ATP proliferation assay

**Figure 1:**
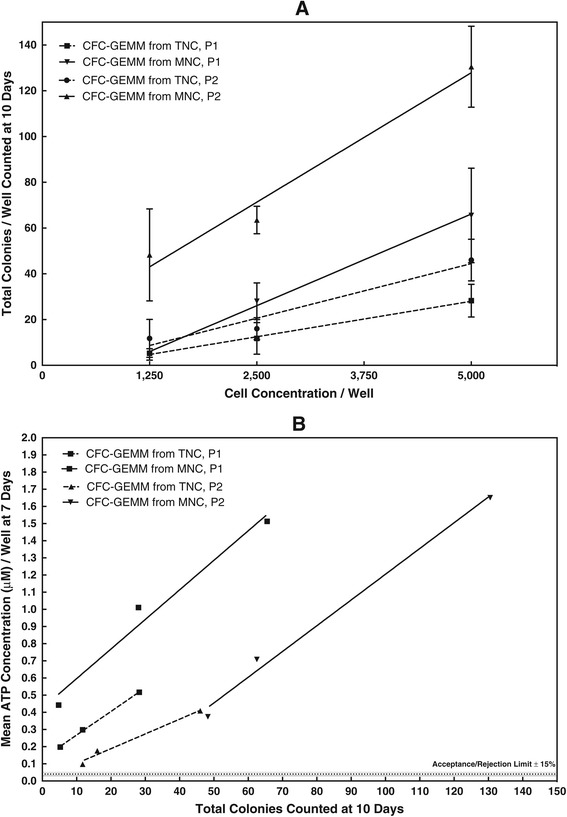
**Correlation between the CFU Differentiation and Proliferation Assays for TNC and MNC Cord Blood Fraction. A.** The Difference in Total CFU Colony Counts for CFC-GEMM Stem Cell Population Enumerated for TNC and MNC Fractions by Two Investigators (P1 and P2)**.** Cell dose response linear regressions. TNC, P1: Slope = 0.0095, r = 0.895. MNC, P1: Slope = 0.0230, r = 0.922. TNC, P2: Slope = 0.0062, r = 0.89. MNC, P2: 0.016, r = 0.91. Probability of the linear regression slopes being equal?. TNC P1 vs MNC P1, P = 0.0009. TNC P2 vs MNC P2, P = 0.0008. TNC P1 vs TNC P2, P = 1.0. MNC P1 vs MNC P2, P = 0.096. TNC P1 vs MNC P2, P = 0.03. TNC P2 vs MNC P1, P = 0.0001. Two-way ANOVA to determine differences between TNC vs MNC and P1 vs P2. TNC P1 vs MNC P1, P = 0.0002. TNC P2 vs MNC P2, P = 0.02. TNC P1 vs TNC P2, P = 0.19. MNC P1 vs MNC P2, P = 0.0001. TNC P1 vs MNC P2, P = 0.0001. TNC P2 vs MNC P1, P = 0.33. **B**. Correlation Between Total CFU Colony Counts for CFC-GEMM Enumerated on Day 10 and CFC-GEMM Proliferation Detected by ATP Bioluminescence on Day 7. Cell dose response linear regressions for 1,250, 2,500 and 5,000 cells/well. TNC, P1: Slope = 0.0137, r = 0.99. MNC, P1: Slope = 0.017, r = 0.99. TNC, P2: Slope = 0.0086, r = 0.99. MNC, P2: Slope = 0.015, r = 0.99.

Two different investigators prepared their own TNC and MNC fractions from two cord blood segments derived from the same lot or unit. The CFU assay was performed at 1,200, 2,500 and 5,000 cells/well to detect the CFC-GEMM stem cell population. The total colony counts were enumerated after 9–10 days of culture by each person and shown in Figure 1A. Although the number of colonies counted was different for each investigator, the correlation coefficients (r) for each cell dose response were high, indicating a high enumeration precision. Furthermore, not only were fewer colonies counted from the TNC fraction compared to the MNC fraction, but the slopes of the cell dose response curves for the TNC fraction were lower than those of the MNC fraction. This implied that the presence of non-colony forming cells in the TNC fraction was impairing the clonal growth of the stem cells.

In parallel with the CFU assay, the same investigators using the same cell preparations and dilutions also prepared an ATP proliferation assay. The cells were cultured for 7 days and the ATP concentrations measured after the assay had been calibrated and standardized as described in the Method section. Figure 1B shows how the total colony counts from the CFU assay correlate directly with ATP concentration values. Like the results from the CFU assay, not only were the correlation coefficients of the cell dose response linear regressions high, but the cord blood TNC fraction produced lower ATP concentrations with a concomitant lower slope for the linear regressions than the MNC fraction. Lower colony counts combined with a decreased slope for the cell dose response, indicated that the TNC fraction affects assay sensitivity caused by other cell impurities. This implies that the lower stem cell response from a TNC fraction might not allow high-quality UCB units to be saved for patient use.

In Table 2, the CFU and ATP values are calculated as 1 × 10^5^ cells and total cells in the segment. For both the number of colonies counted and the ATP concentration measured, the MNC fraction produced higher values than the TNC fraction, although the CFU total colonies for the TNC fraction were not significantly different. In addition, the %CVs for the TNC fraction was approx. 30%, while those for the MNC fraction were less than 10%.

**Table 2:** **Calculation of total primitive hematopoietic stem cell (CFC-GEMM) activity based on the number of colonies and ATP concentrations at 2,500 cells**

	**CFC-GEMM at 1 × 10** ^**5**^ **cells**	**Total CFC-GEMM**
Colony number in TNC fraction	800 ± 242	37,840 ± 11,424
Colony number in MNC fraction	3,175 ± 300	49,085 ± 4,279
ATP (μM) in TNC fraction	8.8 ± 2.6	416 ± 125
ATP (μM)in MNC fraction	35.4 ± 3.3	673 ± 47

Calculation of total CFC-GEMM was performed as described by Page et al. [10] and was based on a recovery of 29.03% after density gradient centrifugation. This recovery was similar to that obtained by Basford et al. [30].

**Statistics for CFU Assay.**

TNC fraction vs MNC fraction: CFU/10^5^, P = <0.0001; Total CFU, P = 0.15.

**Statistics for ATP Assay.**

TNC fraction vs MNC fraction: ATP/10^5^, P = <0.0001; Total ATP, P = 0.024.

### The effect of cell dose and incubation time on the cord blood stem cell response

**Figure 2:**
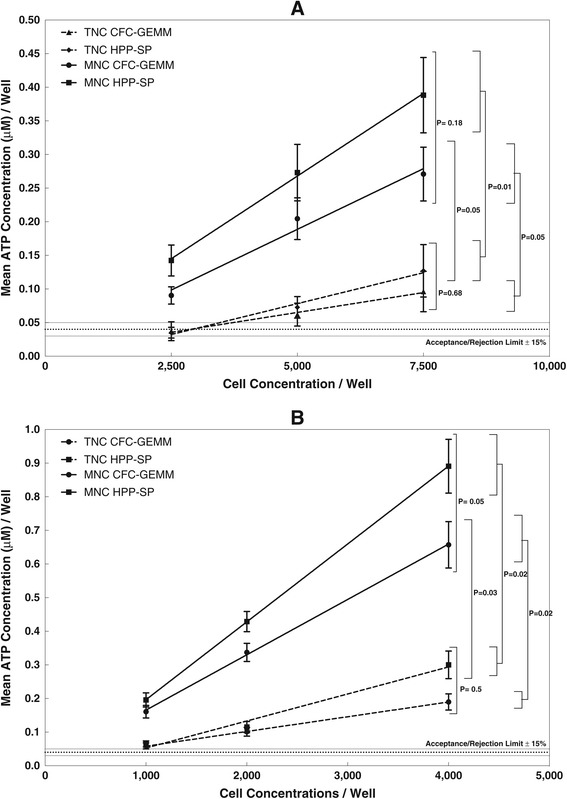
**Optimization of Cell Dose and Incubation Time to Determine the Cord Blood Stem Cell Response for TNC and MNC Fractions. A**. Proliferation Potential of CFC-GEMM and HPP-SP Present in Either TNC or MNC Cultured at High Cell Concentrations (2,500, 5,000, 7,500 cells/well) for 5 Days. Dotted lines represent the TNC fraction, while solid lines represent the MNC fraction. Cell dose response linear regression parameters for N = 22. TNC, CFC-GEMM: Slope = 1.18 × 10^-5^, r = 0.99. TNC, HPP-SP: Slope = 1.84 × 10^-5^, r = 0.99. MNC, CFC-GEMM: Slope = 3.69 × 10^-5^, r = 0.99. MNC, HPP-SP: Slope = 4.92 × 10^-5^, r = 1.0. Probability that the slopes of the linear regression curves are different. TNC CFC-GEMM vs TNC HPP-SP, P = 0.1. MNC CFC-GEMM vs MNC HPP-SP, P = 0.007. TNC CFC-GEMM vs MNC CFC-GEMM, P = 0.02. TNC HPP-SP vs MNC HPP-SP, P = 0.05. Two-way ANOVA between the different response curves shown on the graph as P values. **B**. Proliferation Potential of CFC-GEMM and HPP-SP Present in Either TNC or MNC Cultured at Low Cell Concentrations (1,000, 2,000 and 4,000 cells/well) for 7 Days. Dotted lines represent the TNC fraction, while solid lines represent the MNC fraction. Cell dose response linear regression parameters for N = 41. TNC, CFC-GEMM: Slope = 4.38 × 10^-5^, r = 1.0. TNC, HPP-SP: Slope = 8.04 × 10^-5^, r = 0.99. MNC, CFC-GEMM: Slope = 0.00016, r = 1.0. TNC, HPP-SP: Slope = 0.00023, r = 1.0. Probability that the slopes of the linear regression curves are different. TNC CFC-GEMM vs TNC HPP-SP, P = 0.34. MNC CFC-GEMM vs MNC HPP-SP, P = 0.0008. TNC CFC-GEMM vs MNC CFC-GEMM, P = 0.03. TNC HPP-SP vs MNC HPP-SP, P = 0.02. Two-way ANOVA between the different response curves shown on the graph as P values.

Figure 2 shows the combined results from 22 cord blood segments each prepared as both a TNC and MNC fraction and tested as 2,500, 5,000 and 7,500 cells/well with a 5 day incubation (Figure 2A) and 41 cord blood segments tested at 1,000, 2,000 and 4,000 cells/well with a 7 day incubation (Figure 2B). For all cord blood segments, the proliferation potential for both the HPP-SP and CFC-GEMM stem cell populations were measured.

The results demonstrate that low cell doses combined with a longer incubation time of 7 days produce a more sensitive assay than higher cell doses cultured for only 5 days. For both the TNC and MNC fractions, the HPP-SP exhibits a steeper slope than that for CFC-GEMM, indicating that the former has a greater proliferation potential, is more primitive and therefore more potent than the latter stem cell population. More important, however, is the observation that the TNC fraction produces “stunted” proliferation for both stem cell populations compared to the MNC fraction (cf Figure 1). This effect is directly related to “masking” of the rare stem cell populations by other cell impurities in TNC fraction.

Table 3 shows the combined CVs for the TNC and MNC for the cord blood segments analyzed at low cell concentrations after 7 days of incubation. The results demonstrate a significant amount of variance between the samples. The variance between the stem cell populations in the MNC fraction is between 30 and 50%, while the variance for both stem cell populations within the TNC fraction is not only less constant, but in some cases, more than twice that of the respective MNC population.

**Table 3:** **Mean coefficients of variation for stem cell populations derived from TNC and MNC fractions tested at low cell doses (1,000, 2,000 and 4,000 cells/well) for 7 days**

**Cell concentration / well**	**TNC CFC-GEMM**	**MNC CFC-GEMM**	**TNC HPP-SP**	**MNC HPP-SP**
1,000	100.1%	55.0%	88.8%	55.3%
2,000	85.9%	37.3%	127.6%	51.8%
4,000	101.3%	47.2%	89.6%	47.6%

### Comparison between segments from the same lot

**Figure 3:**
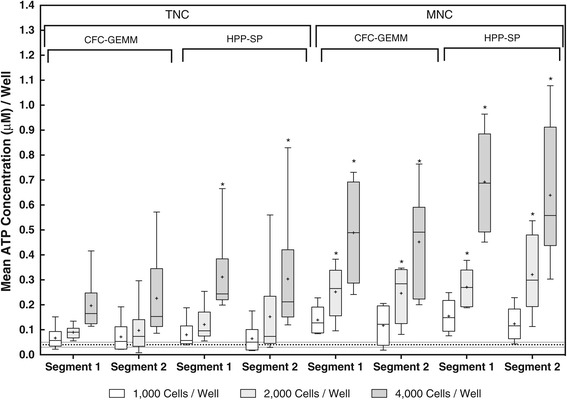
**Comparison Between Paired UCB Segments.** The results represent the combined data from 12 segment pairs from different UCB lots. Segments were randomly allocated as Segment 1 and 2. For each segment, two stem cell populations were measured from both the unseparated, TNC and separated, MNC fractions. The results are provided as box and whisker plots for each cell dose (1,000 cells/well, clear box; 2,000 cells/well, light grey box; 4,000 cells/well, dark grey box) for a total of 12 paired segments. The top and bottom of the box represent the 25 and 75 percentiles. The horizontal line and “+” sign within the box represent the median and mean of the results, respectively. The whiskers (error bars) represent the 5 and 95% confidence intervals. The asterisk (*) represents results from a two-way ANOVA showing a statistical difference (P =< 0.05) of the respective cell dose compared to the CFC-GEMM, TNC, Segment 1 sample. The dotted horizontal lines at the bottom of the graph represent the acceptance/rejection levels ± 15% for cell proliferation measured by ATP bioluminescence.

Segments were analyzed in pairs from 12 different UCB lots. This allowed the stem cell response from each segment to be directly compared with each other. Each segment from a pair was processed by different people using the same methodology. Bland-Altman statistics demonstrated that the results obtained were not due to differences in methodology. Therefore, the differences between the segment pairs were due to the different composition of the cells between the two segments from the same unit. Figure 3 shows the combined box and whisker plots for 3-point cell dose response linear regression curves for each stem cell population detected in each of the two post-thaw, unseparted (TNC) and separated (MNC) segments. The details of the box and whisker plots are given in the legend. The asterisks show which groups of data were significantly different (P = < 0.05) from TNC, CFC-GEMM segment 1, according to a two-way ANOVA.

The only groups that were statistically different within the TNC set of samples were the HPP-SP stem cell population in both segments 1 (P = 0.003) and 2 (P = 0.013). The lack of statistical difference between segments 1 and 2 within the TNC set is due to the low ATP concentrations and the high variances produced by this set of data. All the MNC data demonstrated greater ATP concentrations than the TNC data and therefore significant differences were seen for both segments 1 and 2 at the 2,000 and 4,000 cell/well levels for both stem cell populations. Also noteworthy was the observation that when linear regression curves were fitted to the TNC data from segments 1 and 2 for each of the stem cell populations, the results showed either no correlation or a negative correlation, thereby indicating a difference between the segments of the same unit (data not shown) [38].

### The relationship between cord blood segment and unit

Usually, between two and four contiguous segments are produced and cryopreserved with the UCB unit. One or more of the segments are used primarily for HLA confirmatory testing, but if sufficient cells are available, viability, CD34 and even a CFU assay may also be performed. The object is to confirm that the cells in the segment represent those in the unit and therefore should provide some assurance that the unit can be released and that the cells will perform as expected. Reports have shown that the segment is representative of the sample [39], but others have a shown a discrepancy with respect to the position of the segment in relation to the unit [40]. To test whether the segment is a true representation of the UCB unit, several units with attached segments were examined in parallel. After thawing, an aliquot of the unseparated, TNC fraction was removed from both the segment and the unit. A cell count, viability, and a 3-point cell dose response for CFC-GEMM determined by the CFU assay and CFC-GEMM and HPP-SP measured using ATP bioluminescence. The remaining cells from the segment were processed to a MNC fraction. All of the remaining cells from the unit were divided equally and also processed to a MNC fraction, but by different investigators.

**Figure 4:**
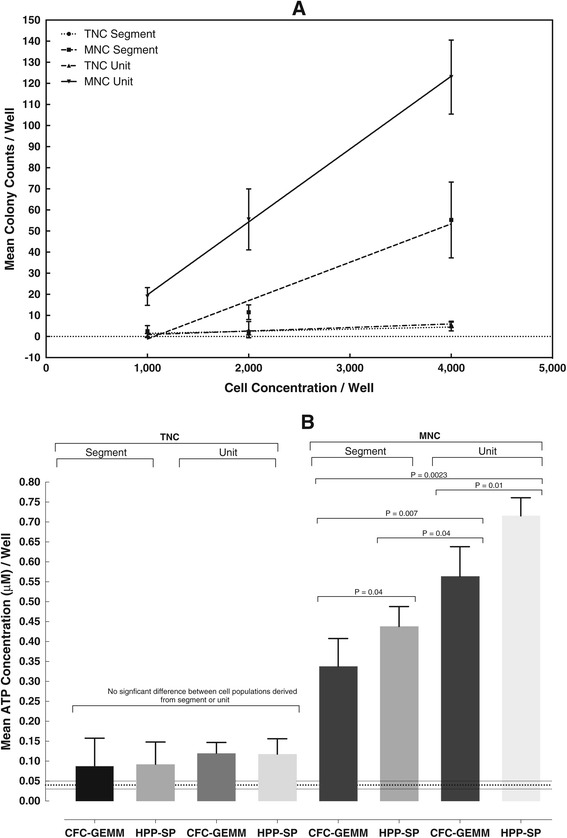
**Stem Cell Response between Cord Blood Segment and Unit for TNC and MNC Fractions. A**. An Example of CFU Results for the CFC-GEMM Stem Cell Population from a Unit and Segment. Closed circles, dotted line; TNC segment: Slope = 0.001, r = 0.92. Closed triangles, dotted and dashed line; TNC unit: Slope = 0.0017, r = 0.98. Closed squares, dashed lines; MNC segment: Slope = 0.018, r = 0.99. Inverted triangles, solid line; MNC unit: Slope = 0.035, r = 1.0. **B**. Difference in Cell Purity and Stem Cell Population Response for UCB Segments and Units. Results are for ATP bioluminescence stem cell proliferation assays at 4,000 cells/well and statistically comparing different parameters using a two-way ANOVA. The horizontal dotted lines at the bottom of the graph indicate the acceptance/rejection limits of the assay ± 15%. None of the values from TNC fractions were significantly different from each other. All ATP concentrations obtained from MNC fractions were highly significant from the TNC values (from P = 0.01 for CFC-GEMM from the MNC segment to P = 0.0002 for HPP-SP from the MNC unit.). The statistical differences (P values) between stem cell populations and segment and unit for MNC fractions are shown on the graph.

Table 4 shows the differential composition of the TNC and MNC fractions for both the segment and the unit. The same considerations described for Table 1 also existed for both the segment and unit in that a considerable number of cells were removed or lost. Despite the low efficiency of the method (which would be considerably improved using automated methods), it remains clear that the post-thaw, TNC fraction contributes to a significant underestimate of the stem cell activity in both the segment and the unit. Figure 4A shows a typical CFU assay for CFC-GEMM and Figure 4B shows the combined results in which CFC-GEMM and HPP-SP stem cell populations (ATP assay) from TNC and MNC fractions were compared between the segment and the unit. The results can be summarized as follows. (1) Both CFU and ATP bioluminescence exhibit the same pattern of results with the TNC fractions showing a lower response than the MNC fractions. (2) The results from up to 9 separate MNC fractions from the unit demonstrated a separate clustering of results for both the CFC-GEMM and HPP-SP stem cell populations, with the latter generally showing a higher slope than the CFC-GEMM population (see (5) below). (3) Using two-way ANOVA, there was no statistical difference between stem cell populations derived from the segment or unit TNC fractions (Figure 4B). This is because the ATP concentrations were too low and the variances too high to allow differences to be distinguished. This might imply that the “null hypothesis” is correct and that for TNC fractions, the segment represents the unit. (4) ATP concentrations from MNC fractions were 3–7 fold greater than those from TNC fractions. Thus, the TNC segment and unit, both provide stem cell response values that severely underestimate the capability of the cells in the unit. Not only was there a statistical difference between the TNC and MNC fractions for both stem cell populations from the segment and unit, but also between MNC-derived stem cell populations from the segment and unit. This was also seen when total cell concentrations were taken into account (Table 5). (5) In some cases, the cell dose response slopes for both stem cell populations derived from the MNC fraction for the segment and unit were statistically parallel, indicating a very low number or lack of primitive lympho-hematopoietic stem cells. This observation implies the absence of cells that could provide long-term engraftment and reconstitution (data not shown). (6) The response of the stem cells in the MNC fraction of the unit provides a more realistic indication of the unit capability. (7) It appeared that the further the segment was from the unit bag, the greater the discrepancy in the stem cell response between the segment and the unit.

**Table 4:** **Comparison of differential counts for the unit and segments post-thaw, unseparated, TNC and separated, MNC fractions**

**Segment cell composition**	**TNC fraction unit (Mean ± St.Dev)**	**MNC fraction unit (Mean ± St.Dev)**	**TNC fraction segment (Mean ± St.Dev)**	**MNC fraction segment (Mean ± St.Dev)**
RBCs (× 10^12^/L)	1.64 ± 0.5	0.5 ± 0.42	0.94 ± 0.3	0.7 ± 0.4
Hematocrit (%)	28.5 ± 9.2	9.0 ± 4.8	15.5 ± 1.0	8.9 ± 6.4
Platelets (× 10^9^/L)	279.0 ± 67.9	35.8 ± 34.4	183 ± 16.3	46.3 ± 6.7
White blood cells (× 10^9^/L)	20.6 ± 6.0	2.5 ± 2.7	12.1 ± 3.3	3.1 ± 0.3
Granulocytes (× 10^9^/L)	12.6 ± 4.5	0.1 ± 0.14	1.0 ± 0.2	0.1 ± 0.05
Lymphocytes (× 10^9^/L)	15.5 ± 1.8	2.4 ± 0.5	9.2 ± 2.6	2.8 ± 0.3

**Table 5:** **Calculation of total stem cell activity between the umbilical cord blood segment and unit based on the number of colonies and ATP concentrations at 2,000 cells/well**

**CFU assay**				
	**CFC-GEMM at 1 × 10** ^**5**^ **cells**	**HPP-SP at 1 × 10** ^**5**^ **cells**	**Total CFC-GEMM**	**Total HPP-SP**
Colonies – TNC Segment	475 ± 96	ND	6,835 ± 1,374	ND
Colonies-MNC Segment	2,275 ± 433	ND	13,533 ± 2,005	ND
Colonies – TNC Unit	487.5 ± 165	ND	8,141 ± 2,759	ND
Colonies – MNC Unit	2,400 ± 248	ND	23,624 ± 1,617	ND
**ATP Bioluminescence Proliferation Assay**				
	CFC-GEMM at 1 × 10^5^ cells	HPP-SP at 1 × 10^5^ cells	Total CFC-GEMM in the Unit	Total HPP-SP in the Unit
ATP (μM) - TNC Segment	22.0 ± 15.1	24.1 ± 20.0	317 ± 217	347 ± 288
ATP (μM) – MNC Segment	58.5 ± 6.5	73.8 ± 2.4	494 ± 44	597 ± 16
ATP (μM) – TNC Unit	29.0 ± 8.6	33.9 ± 11.1	8,732 ± 2,572	10,180 ± 3,335
ATP (μM) – MNC Unit	88.2 ± 11.9	107.2 ± 11.5	13,679 ± 1,440	17,991 ± 1,396

ND = not done.

**Statistics for CFU assay.**

TNC segment vs MNC segment: Colonies/10^5^, P = 0.76; Total colonies, P = 0.17.

MNC unit vs MNC unit: Colonies/10^5^, P = 0.57; Total colonies, P = 0.013.

**Statistics for ATP assay.**

TNC segment vs MNC segment for CFC-GEMM: ATP/10^5^, P = <0.0001; Total ATP, P = 0.024. For HPP-SP: ATP/10^5^, P = <0.0001; Total ATP, P = 0.0032.

TNC unit vs MNC unit for CFC-GEMM: ATP/10^5^, P = <0.0001; Total ATP, P = 0.01. For HPP-SP, ATP/10^5^, P = <0.0001; Total ATP, P = 0.032.

Table 5 shows the calculated colony number and ATP concentrations for the two stem cell populations detected in the TNC and MNC fractions of both the segments and unit. CFU colony counts based on 1 × 10^5^ cells were not significantly different between the TNC segment and unit (P > 0.05) and MNC segment and unit (P > 0.05). Based on the CFU output, this might imply that the segment represented the unit. However, the calculated total CFC-GEMM content for the MNC fraction does not confirm this. When the proliferation ability of the stem cells was examined based on 1 × 10^5^ cells, the TNC fractions in the segments and unit were also similar, but the values for the MNC fractions were significantly different between the segment and unit for both stem cell populations. These differences were also observed when total UCB unit values were calculated. These results illustrate the need for caution in assuming that the segment is a true representation of the unit.

### Comparison between umbilical cord blood samples from two cord blood banks

The 41 sample segments tested at low cell doses for 7 days, were obtained from two cord blood banks (CBB), 12 from CBB1 and the remainder from CBB2. Table 6 shows the mean and SEM of the combined cell dose response data for each of the stem cell populations determined from the TNC and MNC fractions for each cord blood bank. It would appear that CCB1 produces sample segments in which the overall stem cell proliferation ability (determined at 4,000 cells/well), is greater than CCB2. However, also shown are the percent CVs. These values indicate that for both CBBs, the TNC fraction produces lower values and higher variances compared to the MNC fraction. Comparing the results between CBBs using ANOVA produced differences in results for stem cell populations detected only at the higher cell dose levels. In addition, it was only at these high cell dose levels that significant differences were seen between TNC and MNC fractions.

**Table 6:** **Comparison between umbilical cord blood samples from two cord blood banks**

		**Cord Blood Bank 1 (N = 12)**		**Cord Blood Bank 2 (N = 29)**	
**Cell concentration**	**Cell population**	**TNC (mean ± SEM %CV)**	**MNC (mean ± SEM %CV)**	**TNC (mean ± SEM %CV)**	**MNC (mean ± SEM %CV)**
1,000	CFC-GEMM	0.09 ± 0.03, 91.9%	0.238 ± 0.06, 64.5%	0.057 ± 0.01 91.7%	0.122 ± 0.01, 60.6%
	HPP-SP	0.099 ± 0.03, 99.7%	0.323 ± 0.06, 56.5%	0.06 ± 0.01, 90.3%	0.146 ± 0.02, 58.7%
2,000	CFC-GEMM ^(+)^	0.165 ± 0.04, 74.2%	0.544 ± 0.1, 57.6% [1]	0.085 ± 0.01, 86.3%	0.25 ± 0.02, 46.1% [3]
	HPP-SP ^(*)^	0.21 ± 0.06, 79.6%	0.723 ± 0.13, 56.2% [2]	0.126 ± 0.03, 111.0%	0.308 ± 0.03, 79.7% [3]
4,000	CFC-GEMM ^(*)^	0.284 ± 0.08, 87.4%	0.887 ± 0.16, 58.2% [2]	0.177 ± 0.03, 81.9%	0.499 ± 0.05, 49.0% [4]
	HPP-SP ^(*)^	0.396 ± 0.1, 73.0%	1.352 ± 0.2, 47.4% [2]	0.293 ± 0.05, 87.8%	0.704 ± 0.06, 45.9% [4]

Samples from CBB 1 used Sepax technology.

Samples from CBB 2 used AXP technology.

(+) represents 2,000 cells/well between MNC from CBB1 vs CBB2, P = 0.05.

(*) represents MNC from CBB1 vs CBB2, P < 0.001.

[1] represents TNC vs MNC from CBB1, P = 0.05.

[2] represents TNC vs MNC from CBB1, P < 0.001.

[3] represents TNC vs MNC from CBB2, P = 0.05.

[4] represents TNC vs MNC from CBB2, P < 0.001.

### The relationship between viability and ATP bioluminescence

**Figure 5:**
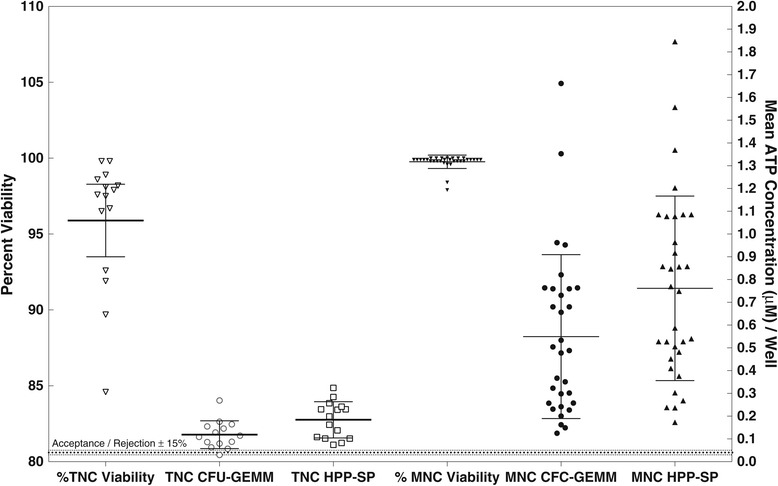
**Dye Exclusion Viability Versus Metabolic Viability and Stem Cell Proliferation for TNC and MNC Fractions.** Percent viability, detected by 7-AAD and flow cytometry, is shown on the left Y-axis, while mean ATP concentration (μM) / well is given on the right Y-axis. The results show individual measurement as symbols. The mean (dark horizontal line) ± 95% confidence intervals are provided as error bars. Values outside the top and bottom bars indicate outliers.

Table 1 showed similar viability values between TNC and MNC fractions. Indeed, during the course of these studies, it became clear that the percent viability, detected by 7-AAD, did not correspond to the metabolic viability detected when either the CFC-GEMM or HPP-SP were stimulated. It is important to distinguish between these two types of viability measurement. Dye exclusion viability using 7-AAD and flow cytometry detects membrane integrity, whereas ATP (and other metabolic biochemical markers such as MTT) require active mitochondria and detect metabolic and cellular integrity and therefore provides a functional measure of viability. Figure 5 demonstrates that the high dye exclusion viability obtained from TNC fractions does not correspond to the low proliferation found with either stem cell population. Indeed, even the viabilities obtained for the MNC fractions, which were close to 100%, did not correlate with stem cell proliferation. These results indicate that using dye exclusion viability as a means of predicting cell growth can produce high, false positive values and therefore can significantly influence the interpretation of the results.

## Discussion

The results presented in this communication demonstrate that rare stem cell populations can be detected and quantitatively measured in very small volumes of UCB sample segments. The results also show that UCB cell preparation purity and viability can have a significant influence on detecting specific cell populations as well as how they are measured. Compared to the MNC fraction, the cell impurities in the TNC fraction are not only responsible for producing high variability, but also masking the presence of the rare stem cells populations. This, in turn, significantly underestimates, both the quality and potency of the stem cells in the UCB segment and unit. The TNC fraction is considered one of the basic parameters upon which several important assumptions are made, including whether the unit will be stored for transplantation purposes and uploaded to a cord blood inventory, the quality of the unit, its release for transplantation purposes and correlation with clinical outcome [41]. The present results not only call into question the use of pre-freeze testing to represent the post-thaw unit, but many of the assumptions regarding how the quality and potency of a cord blood unit can be determined prior to transplantation.

The ATP bioluminescence signal detection system is the most sensitive, non-radioactive readout available for cell viability and function [42]. Previous studies had demonstrated the correlation between CFU colony counts and the measurement of iATP as a biochemical marker for hematopoietic cell proliferation [43]. The present results again demonstrate this correlation for UCB stem cells not only in sample segments, but also in the whole unit. This verification of one assay against the other substantiated the use of the ATP bioluminescence, CFU-alternative assay to perform this study. That the ATP bioluminescence stem cell proliferation assay is calibrated and standardized, thereby providing an internal proficiency test every time samples are processed, and has been validated according to regulatory guidelines [33], lends further credibility to the results.

Cell viability and nucleated cell counts are two of the most basic laboratory parameters required to perform most cell-based assays. Dye exclusion methods such as trypan blue, propidium iodide, acridine orange and 7-AAD are regularly used to measure cell viability for many applications. The use of 7-AAD is usually combined with the measurement of CD34 by flow cytometry to determine the number of viable CD34^+^ cells in the UCB segment and/or unit [44,45]. It has always been assumed that dye exclusion viability provides a rapid and reliable measure of live/dead cells in the suspension. The possibility that a viability measurement may produce false positive results is of particular concern, since this has many serious repercussions. These include the decision to permanently store a UCB unit, add it to a cord blood inventory, and use the unit for transplantation purposes.

The TNC count has been used in hematopoietic cell transplantation for decades. By definition, the TNC fraction contains virtually all nucleated cells, including granulocytes, as well as varying concentrations of platelets, red blood cells and other cell types. Many of these are lysed or killed during the cryopreservation process. It is not known whether stem cells undergo the same fate. Nevertheless, the post-freeze TNC fraction still contains high levels of cell impurities. These impurities do not contribute to the engraftment process and, as demonstrated in both the segment and unit, actually hinder the detection of the stem cells responsible for engraftment by diluting and “masking” their presence. This effect has also been observed using the CFU assay [24]. Although density gradient centrifugation is a simple and rapid purification step from the TNC to MNC fraction and was perfectly suitable to test the hypothesis proposed for these studies, it is certainly not optimal for the rapid and cost-effective processing of large numbers of UCB units. Automated, “closed” systems have been available to further purify the cells in UCB units for some time [22,24], but are rarely used, since cord blood banks normally perform plasma and red blood cell reduction. It would appear that storing UCB units as MNC fractions instead of TNC fractions would provide more accurate and reliable results that would be more relevant to the transplant physician, since at the very least, it affects both the “quality” and potency of the UCB unit.

What constitutes a high-quality, and high potency UCB unit, is still a matter of debate [46-48]. The non-binding recommendations of the FDA [49] provide values for the minimum TNC, viability and viable CD34^+^ content as measures of purity and potency. For processing, a UCB unit usually requires a TNC count of at least 1 × 10^9^ cells [50], with UCB units containing greater than 1.79 × 10^9^ TNC being preferred by transplantation centers [26,51]. As demonstrated here, increased cell dose correlates with increased ability to detect and measure stem cell proliferation in the unit. Therefore, the higher the TNC count, the greater the proportion of stem cells in the unit and the higher the probability of engraftment. This is because engraftment is dependent upon the ability of the stem cells to proliferate. If stem cell proliferation cannot be adequately measured because impurities in the TNC fraction mask, impair and underestimate their functionality, then the quality of the UCB unit cannot be accurately and reliably determined. By definition, the CFU-GEMM stem cell population is characterized by the presence of granulocytes and macrophages, erythroid cells and megakaryocytes within a single colony. The identification of these cell types by flow cytometry after stimulation of the CFC-GEMM population in an ATP bioluminescence assay was demonstrated previously [31]. However, most methylcellulose formulations do not contain TPO required to potentiate stem cells and stimulate megakaryopoiesis. As a result, the population actually detected by this reagent is more mature and therefore correlates to a greater degree with CD34 and GM-CFC [46], than that detected in the present studies. For normal UCB testing, the CFU assay is used primarily to determine the presence of granulocyte-macrophage (GM) progenitor cells as a predictor of granulocyte neutrophil production and an indicator of time to neutrophil engraftment [10]. Since this is part of the early reconstitution process and downstream from stem cell engraftment, detection of GM-progenitor cell growth neither predicts nor determines the potential of the stem cells for engraftment [50]. Even though determination of CD34 membrane expression is considered a “stem cell marker”, it is far from specific [24], and provides no information on stem cell functionality. The CD34 marker was not determined in these studies because (a), it would have depleted cell numbers for measuring cell proliferation, and (b), previous studies had shown that CD34 did not correlate with ATP, since it is not a stem cell marker [32,35]. Taken together, it is necessary to seriously question the notion that a UCB unit can be of high-quality and potency when (a) the sample being tested is impure, and (b), that the presence and functional properties of stem cells in the product are not detected and measured to predict engraftment potential.

Throughout these studies, a minimum 3-point cell dose response was performed for all segments and units tested. This allowed two parameters of stem cell proliferation to be determined simultaneously. The first was the ability of the stem cells to proliferate at a specific cell dose, and at the time the sample was thawed. This parameter is proliferation ability or status and is equivalent to stem cell “quality”. The second is proliferation potential, a key property of stem cell primitiveness, and determined from the slope of the stem cell dose response linear regression [34]. The steeper the slope of the cell dose response, the more primitive the stem cell population. As shown, the HPP-SP is more primitive than the CFC-GEMM stem cell population. However, proliferation potential correlates with several other parameters. These include stem cell self-renewal capacity and engraftment potential. These parameters are, in turn, directly related to the potency of the stem cell population; the more primitive a stem cell population, the greater its proliferation potential and potency and, therefore, its engraftment potential. It has been shown previously that proliferation potential is a requirement for measuring the potency of the “active” stem cell components [31,32,35-37]. The present results clearly demonstrate that measuring UCB stem cell proliferation is also a requirement if accurate and reliable information is to be accrued and used in the cord blood inventories.

The TNC count, viability, viable CD34^+^ count and the total number of colonies or number of GM colonies have been used for decades to characterize UCB samples and equate the results to the unit. This was the reason for hypothesizing that using the TNC fraction from UCB segments and units did not have to be further purified in order to accurately, reliably and reproducibly measure the stem cells. The data presented above clearly reject this hypothesis. Cord blood segments and units that contain varying degrees of cell impurities cannot be used to determine UCB stem cell quality or potency. It might be argued that since hematopoietic stem cell transplantation has been a routine procedure for decades using essentially the same tests and assays, there is no reason to characterize and measure the stem cells prior to infusion into the patient. However, it is now obvious that several basic assumptions upon which UCB transplantation have been based, have now been called into question. Umbilical cord blood transplantation continues to deal with long engraftment times (usually 20–25 days) [8-10], and a high rate of graft failure (20-24%) [8,10,52], both of which are detrimental to the patient. Umbilical cord blood is being used for numerous clinical applications and virtually all use the same basic tests and assays and therefore the same basic assumptions. As argued by other authors [27,28], improvements can and should be made. The present data clearly substantiates those arguments, but also provides simple and substantive methods for improvement that might help reduce engraftment times, graft failure, and ultimately benefit the patient.

## Conclusions

According to the NMDP [53], more than 25,000 cord blood unit stem cell transplants have been performed worldwide. All have been characterized using TNC, viability, viable CD34 content and the CFU assay, but virtually none have been analyzed to ensure that the stem cells responsible for engraftment exhibit high functional quality and potency. Using a highly sensitive and standardized ATP bioluminescence stem cell proliferation assay, it is shown that the TNC fraction, upon which many of the decisions are made, from cord blood storage to release of a unit for transplantation purposes, not only dilute and mask the stem cells responsible for engraftment, but can underestimate the quality and potency of the UCB unit. The results call into question many of the premises that are being used in cord blood banking and processing. These include differences between individual cord blood sample segments prepared at the time the unit is processed and used for confirmatory testing, assuming the segment is a true representation of the cord blood unit and high false positive viability values that do not correlate with the metabolic viability and functionality of the stem cells in the segment or unit. The results demonstrate that the more purified MNC fraction will provide more precise and accurate measurements of stem cell properties that can be used to better define the quality and potency of the cord blood unit prior to use. Indeed, the results imply that cord blood units should be stored as MNC rather than TNC preparations. In addition, although testing must be performed on the pre-freeze unit to determine stem cell suitability for storage and later use, specific stem cell quality and potency determinations should be performed, and the information uploaded to cord blood inventories, on samples of cells shortly after cryopreservation. This would provide more relevant details on the stem cell status that better represent the unit when it is eventually selected by a transplantation center to treat a patient.
